# X-Linked Gusher Disease DFNX2 in Children, a Rare Inner Ear Dysplasia with Mixed Hearing and Vestibular Loss

**DOI:** 10.3390/audiolres13040052

**Published:** 2023-08-04

**Authors:** Soumit Dasgupta, James Hong, Rhyanna Morris, Javed Iqbal, Amy Lennox-Bowley, Jeyasakthy Saniasiaya

**Affiliations:** 1 Alder Hey Children’s Hospital NHS Foundation Trust, Eaton Road, Liverpool L14 5AB, UK; 2 Faculty of Health and Medical Sciences, University of Liverpool, Liverpool L69 3BX, UK; hlshong@liverpool.ac.uk (J.H.); hsrmorr2@liverpool.ac.uk (R.M.); 3 East Lancashire Hospitals NHS Trust, Burnley BB10 2PQ, UK; javed.iqbal2@elht.nhs.uk; 4 Hypatia Dizziness and Balance Centre, Liverpool L1 9ED, UK; amy@hypatiatraining.com; 5 Faculty of Medicine, University of Malaya, Kualalampur 50603, Malaysia; jeyasakthy@um.edu.my

**Keywords:** third window, X-linked gusher, hearing loss, audiovestibular, *POU3F4*

## Abstract

Conductive hearing losses are typically present in disorders of the external/middle ear. However, there is a rare group of inner ear conditions called third windows that can also generate a conductive hearing loss. This is due to an abnormal connection between the middle and the inner ear or between the inner ear and the cranial cavity. X-linked gusher disorder is an extremely rare congenital inner ear dysplastic syndrome with an abnormal connection due to a characteristic incomplete cochlear partition type III and an incomplete internal auditory meatus fundus. The disorder is inherited in an X-linked fashion due to the mutation of the *POU3F4* gene. We present two siblings diagnosed with the condition and their long-term follow-ups. They both presented audiovestibular symptoms and showed progressive mixed losses and bilateral vestibular weakness. They were treated with cochlear implant, digital amplification and with vestibular rehabilitation. Significant others around them were involved in their journey with the medical team, and in both, a very favourable outcome was achieved. This is the first time that we have reported evolving audiovestibular function with vestibular quantification in X-linked gusher disorder and emphasize on the multidisciplinary holistic approach to manage these children effectively.

## 1. Introduction

X-linked gusher disease is a rare genetic disorder that presents a conductive element in hearing loss measured by pure tone audiometry. First identified by Nance in 1971, this entity was considered a congenital stapedial fixation disorder, where during surgery, a perilymphatic gusher was identified [1]. The disorder is characterized by classical radiological appearances based on high-resolution CT scan (HRCT). Phelps et al. in 1991 studied and observed the CT phenotype of an X-linked gusher disorder with three features—bulbous internal auditory meatus (IAM), incomplete separation of the coils of the cochlea from the IAM and wide first and second parts of the intratemporal facial nerve [2]. Subsequently, an absent bony modiolus was identified [3].

The clue that the disorder is genetically inherited was suggested by observing the disorder in males. The gene responsible for the disorder was identified in 1995 [4] as the *POU3F4* gene. The POU family of genes is a family of eukaryotic transcription factors regulating neuroendocrine function, of which the *POU3F4* gene is a key factor for mesenchymal integrity, spiral ganglion functioning, spiral ligament structure and stria vascularis activities [5]. Mutations in this gene lead to an otological phenotype. The gene was mapped to chromosome Xq13-q21.1 in 1988 [6]. The mutation was designated DFNX2 by gene mapping and is the second in a group of five X-linked non-syndromic genetic deafness groups [7].

X-linked gusher disorder mostly, if not always, occurs in males given its mode of inheritance. However, females may be obligatory carriers presenting a milder phenotype [3,8]. There was one case report where typical X-linked gusher-associated radiological findings were detected in a female, suggesting a separate mode of inheritance yet to be identified [9]. X-linked gusher DFNX2 is the most common of the five X-linked non syndromic hearing loss group comprising about 50% [10]. The X-linked group cumulatively constitutes about 1–5% of non-syndromic hearing loss [10]. DFNX2 is very rare [11] and true prevalence is unknown. Given that 1 in 1100 live births will show a congenital bilateral permanent hearing loss, of which 80% are non-syndromic genetic, and X-linked recessive hearing losses account for only 5% of this non syndromic group, of which 50% is DFNX2, the rough prevalence will be 0.0018 per 1100 live births. The condition is defined by the typical cochlear dysplasia [12].

The hearing loss encountered is heterogenous, ranging from moderate to severe and profound conductive to sensory cochlear hearing loss, but the mixed variety is the most common [4,13]. The cochlear hearing loss can be intuitively inferred by cochlear dysplasia. The conductive component has been postulated to be due to a third-window effect that essentially arises due to a direct communication between the subarachnoid space in the cranial cavity and the perilymphatic space in the inner ear due to the incomplete cochlear partition and absent modiolus at the fundus of the IAM [14,15]. In a series of rare third-window abnormalities in children, DFNX2 accounted for about a fifth of all rare third-window disorders [11]. Vestibular function quantified with objective vestibulometry was reported only once [11].

In this study, we present two siblings who were diagnosed with the condition and underwent full behavioural/objective audiological and objective vestibular quantification. These siblings were reported earlier as part of a cohort [11] with summarised phenotypes. This paper describes the clinical features, clinical/objective audiovestibular quantification and treatment outcomes of these siblings over a period of time. This paper highlights the importance of audiological surveillance and a vestibular phenotype in a rare disorder of the inner ear generating a conductive hearing loss. To our knowledge, a vestibular phenotype with quantification of vestibular function that includes the suppression head impulse test and long-term audiological management outcomes have not been studied in X-linked gusher disorder in children.

## 2. Methods

Two children out of a total of fifty-three third-window disorders in a tertiary paediatric vestibular centre in the UK over a period of 3.5 years were diagnosed with X-linked gusher disorder, comprising 3.7% of all third-window disorders. Both children were diagnosed with detailed anamnesis, exclusion of other conditions and imaging studies (HRCT/T2 drive MRI). Both would show mutations in the *POU3F4* gene.

Pure tone audiometry following British Society of Audiology standards, tympanometry and acoustic reflexes, transient otoacoustic emissions were performed to quantify hearing with an Aurical Audiometer, Otometrics Zodiac and Otodynamics equipment, respectively.

Videonystagmography (VNG) with and without optic fixation (nystagmus, smooth pursuits and saccades, head shake, head heave, ocular counter roll and the mastoid vibration test), vestibulospinal test battery (VST) with and without foam cushion (Romberg, Unterberger and tandem gait), video head impulse test (vHIT), suppression head impulse test (SHIMP), cervical vestibular-evoked myogenic potential test (cVEMP) and measurement of the subjective visual vertical (SVV) were utilised to quantify the vestibular system. Our own laboratory norms were used when analysing vHIT, SHIMP and cVEMP results (our vHIT VOR gains range from 0.9 to 1 in horizontal canals; 0.6 to 0.8 in vertical canals between the ages 6 and 16 years; up to 20% asymmetry is accepted in SHIMPs with a peak saccadic velocity cut off at 180°/s between ages 4 and 16 years, and our VEMP laboratory norms include asymmetry up to 26% and thresholds of 80 ≥ dBnHL between the ages of 4 and 16 years). The ICS Impulse 2015 and 2019 versions and Neurosoft 2019 software were used for VNG/vHIT/SHIMP and VEMPs, respectively. The first author performed all vestibular tests except the VEMPS, which was performed by one of the co-authors, and consistency was maintained.

We did not perform ocular vestibular-evoked myogenic potentials (oVEMP), as paediatric norms are hardly available, and we are in the process of gathering our own norms. These tests were repeated several times as part of audiovestibular surveillance. All children also underwent neurological, paediatric, cardiological and development assessment as mandated in investigating children with suspected vestibular disorders. The families, the sensory services, the school and significant others were all involved in the journey of the children effectively.

Informed signed consent was obtained from both children and their legal guardian for reporting their conditions to scientific journals. Ethical Committee approval was not required as the study was not classed as research but as a case series with less than 3 subjects.

## 3. Results

### 3.1. Sibling A (SA)

**Figure 1:**
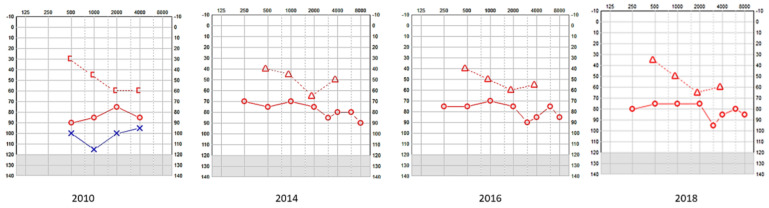
SA—serial pure tone audiometry (PTA) showing profound hearing loss on the left and a mixed fluctuating and progressive hearing loss on the right; the left underwent a cochlear implant and the right received digital amplification. [-Bone conduction right masked; o-Air conduction right; Δ-Bone conduction right unmasked; x-Air conduction left.

This child did not undergo the universal newborn hearing screening as it was in its infancy in the country of study. He was referred due to delayed speech and motor development at the age of 2 years and 3 months and a severe mixed hearing loss was observed in both ears. His tympanometry was bilaterally normal as was stapedial reflex on the right only and his transient otoacoustic emissions were absent bilaterally. He was fitted immediately with digital amplification and monitored twice every year. The hearing loss progressed over the next years, especially on the left ear, becoming profound (Table 1, Figure 1), which necessitated a cochlear implant on that side after 7 years. The right side also showed deterioration, but he refused an implant as he was deriving good benefits from the hearing aid on that side. His speech and communication abilities further improved with speech and language therapy. Speech tests with and without noise were acceptable at age 15 years. He went to a mainstream school and performed well with his academia and developed a good positive insight for the future, looking forward to university life.

**Figure 2:**
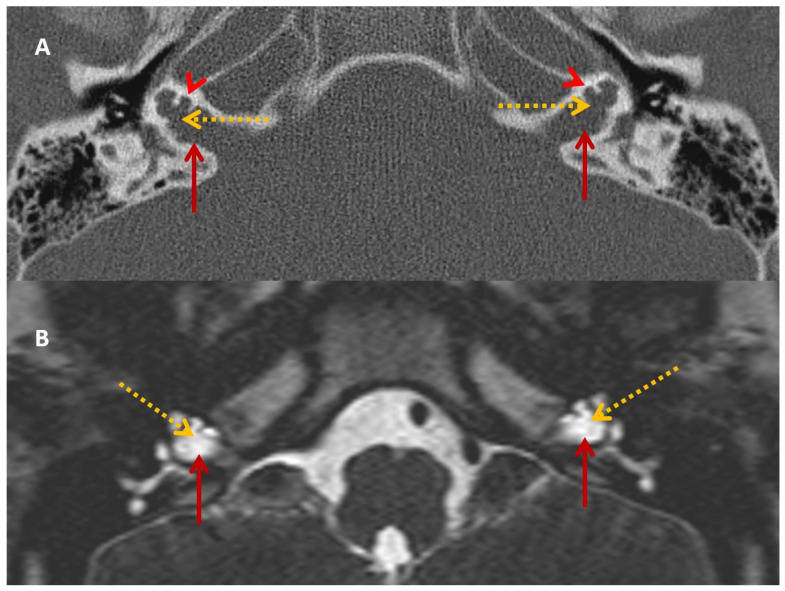
SA—HRCT and MRI. HRCT (**A**) and T2 Drive MRI (**B**) in sibling SA showing bilateral bulbous dilatation involving the fundus of the internal auditory canal (maroon arrow) and bilateral incomplete separation of the basal turn of the cochlea (red arrowhead) from the fundus of the internal acoustic canal (dotted yellow arrow), classical of X-linked gusher disorder.

HRCT and T2 drive MRI demonstrated a bulbous end of IAM with dilatation, absent cochlear modiolus with intact septa, incomplete cochlear partition type III and incomplete IAM fundus gene (Figure 2) that confirmed the diagnosis of an X-linked gusher disorder. He would show the POU3F4 mutation by genetic typing.

**Figure 3:**
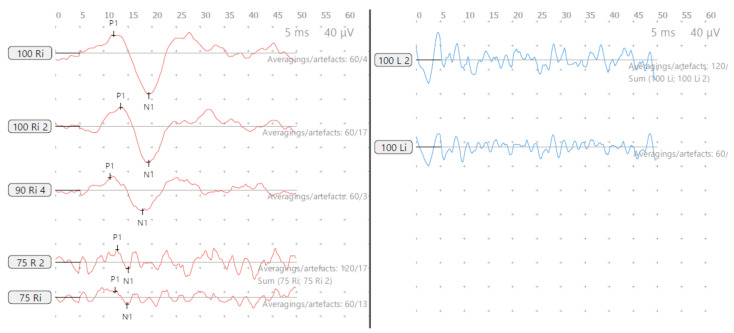
SA—cVEMP age 15 years, absent response left and low threshold on right.

**Figure 4:**
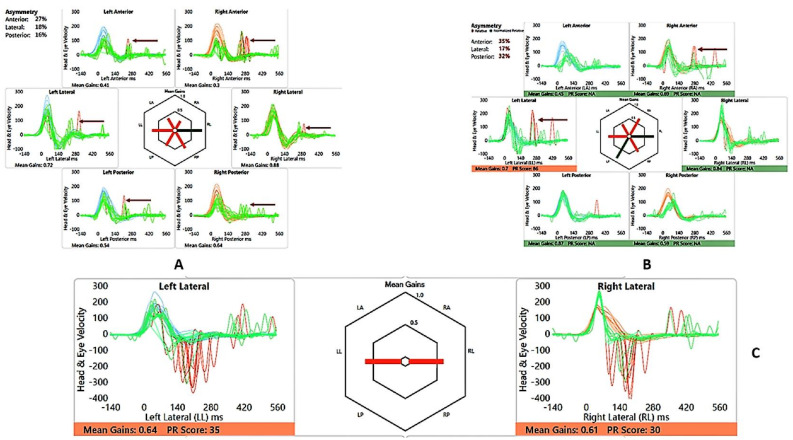
SA—vHIT and SHIMP. (**A**) vHIT age 12 years, note low VOR gain and catch-up saccades (arrow); (**B**) vHIT age 15 years, note recovery of VOR gain and absence and reduction of saccades (arrow); (**C**) SHIMP age 15 years, normal asymmetry and peak saccadic velocities.

He perceived balance problems and was apprehensive in playground activities when he was a toddler. He was fond of dancing but was unable to indulge in the twists/turns that dancing demanded. He was diagnosed with bilateral vestibular dysfunction 12 years after diagnosis, showing an initial six semicircular canal involvement in the vHIT, absent cVEMP on the left with low thresholds on the right, with abnormal VST but normal SHIMP, SVV and VNG (Table 2 and Table 3 and Figure 3 and Figure 4). There was no Tullio’s or Hennebert’s phenomenon in his history or objective testing. Vestibular rehabilitation was provided. His vestibular function improved over the years subjectively and objectively in the vHIT to the point of resuming his dancing and overcoming his apprehension with balance.

### 3.2. Sibling B (SB)

**Figure 5:**
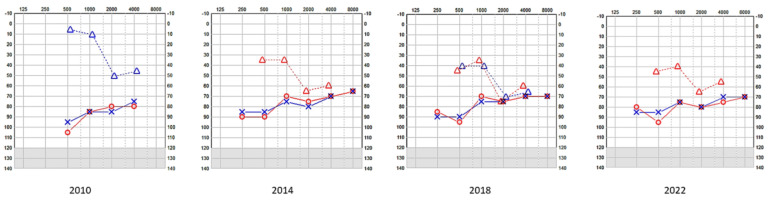
SB—serial pure tone audiometry (PTA) showing bilateral mixed hearing—both ears received digital amplification. o-Air conduction right; Δ-Bone conduction right unmasked; Δ-Bone conduction left unmasked x-Air conduction left.

Sibling B failed his newborn hearing screening and showed a moderate mixed hearing loss on both sides. He was fitted at the age of 6 weeks and was regularly monitored. There was a mild speech delay and he received speech and language therapy. His hearing loss also showed progression and fluctuation and became severe over the years (Table 4 and Figure 5). His tympanometry was normal as were his stapedial reflexes, but transient otoacoustic emissions were absent on both sides. His amplification was continuously revised, and his speech became age appropriate as did his communication skills. Speech tests with and without noise were acceptable at age 12. He too went to mainstream school and performed quite well with his academia. He has good positive insight into the future and wants to pursue a career in audiology as he believes that our input from such an early period has changed his life.

**Figure 6:**
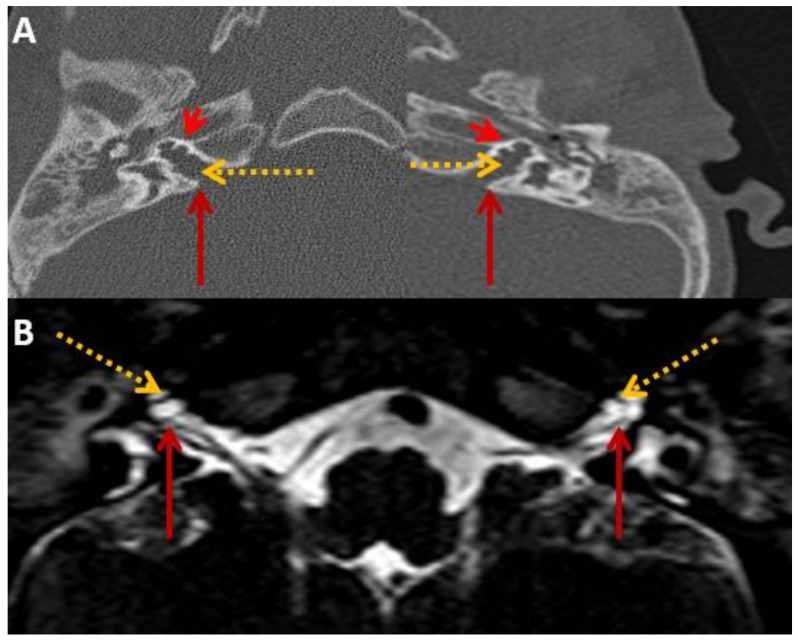
SB—HRCT and MRI. HRCT (**A**) and T2 Drive MRI (**B**) in sibling B showing bilateral bulbous dilatation involving the fundus of the internal auditory canal (maroon arrow) and bilateral incomplete separation of the basal turn of the cochlea (red arrowhead) from the fundus of the internal acoustic canal (dotted yellow arrow) classical of X-linked gusher disorder and similar to SA in Figure 2.

HRCT and T2 drive MRI was identical to his brother (Figure 6), confirming the diagnosis of an X-linked gusher disorder. He would also show the *POU3F4* mutation by genetic typing.

**Figure 7:**
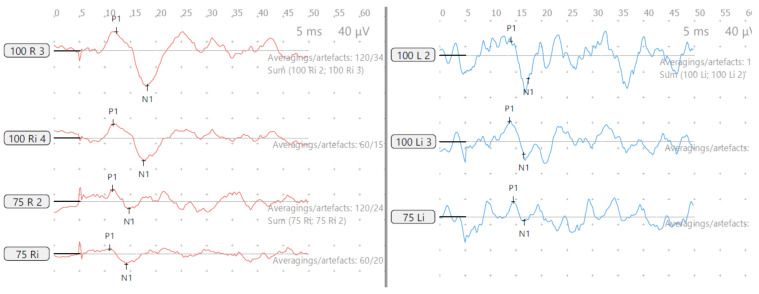
SB—cVEMP age 13 years, reduced amplitudes and low threshold both sides.

**Figure 8:**
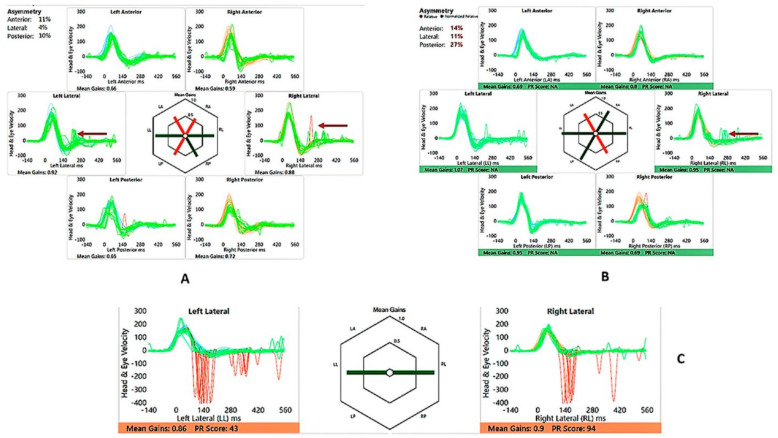
SB—vHIT and SHIMP. (**A**) vHIT age 10 years, note catch-up saccades (arrow); (**B**) vHIT age 13 years, note reduction of saccades (arrow); (**C**) SHIMP age 13 years, normal asymmetry and peak saccadic velocities.

His motor skills were delayed, and like his brother, he was also apprehensive about playground activities. He was diagnosed with bilateral vestibular weakness 10 years after diagnosis (Table 5 and Table 6 and Figure 7 and Figure 8) involving the lateral semicircular canals on the vHIT, reduced amplitudes and lowered thresholds in the cVEMP, with abnormal VST but normal SHIMP, SVV and VNG. He received vestibular rehabilitation. He did not complain of any balance problems since then and has good balance function confirmed by latest/recent objective tests. Of third-window features, he perceived autophony, but there was no Tullio’s or Hennebert’s phenomenon in his history or objective testing. 

## 4. Discussion

X-linked gusher disorder is diagnosed by a characteristic phenotype of congenital progressive mixed hearing loss with normal middle ear function and by a pathognomonic HRCT demonstratable cochlear dysplasia. The disorder is due to a mutation in the *POU3F4* gene with 51 different loci identified [16]. Our siblings fulfilled the criteria to satisfy the diagnosis of an X-linked gusher disorder.

The typical feature of an X-linked gusher disorder in HRCT is an incomplete cochlear partition type III. The *POU3F4* gene in the animal model participates in the remodeling of the otic capsule [17]. A breach in this remodeling leads to the incomplete cochlear partition. There are three types of incomplete cochlear partition [18]. Although the three different varieties resemble each other, the essential difference between the three is in the way the modiolus and the IAM fundus are formed. In type I, the modiolus is completely absent with its interscalar septa; in type II, only the apical modiolus is absent with its septa; in type III, the modiolus is absent but the septa are present. Type II is also associated with dilated vestibular aqueduct [18]. In X-linked gusher disorder, type III is uniquely observed in addition to a dilated internal auditory meatus, incomplete separation of the basal turn of the cochleae from the fundi of the IAC that includes defects in the lamina cribrosa, bulbous fundal end of the IAM and a thin otic capsule [12]. These features make it pathognomonic of the condition. The siblings in this series exhibit these classical features.

Other cochlear structural abnormalities defined in X-linked gusher disorder are an enlarged superior vestibular nerve canal, enlarged labyrinthine facial nerve canal, enlarged singular nerve canal, vestibule with cystic appearance, semicircular canal with cystic appearance, dysplasia of oval window, dysplasia of round window and abnormal stapes [13].

Unlike HRCT, MRI observations in the condition have hardly been reported. T2 MRI in X-linked gusher disorder shows a bulbous internal auditory canal, an incomplete separation of the basal turn of the cochlea with the fundus and a fluid-filled cochlear cavity without the modiolus or the spiral lamina [19]. An MRI also may show a hypothalamic lesion [20]. Our siblings showed these consistent features in their HRCT and MRI to establish a radiological diagnosis but without any brain abnormality.

A typical feature observed in X-linked gusher disorder is a conductive element to the hearing loss that it presents, which was universally observed in all case reports [1,2,3,4,5,6,7,8,9,10,11,12,13,14,15,16,18,19,20,21,22]. Conductive hearing loss not originating from the external or the middle ear can be due to the third-window phenomenon that is generated by an inner ear group of disorders called third-window disorders. Third-window disorders are structural abnormalities in the bony otic capsule that establish a connection between the middle/inner ear or the inner ear/cranial cavity [11]. The Minor group in 1998 identified the first third-window prototype superior semicircular canal dehiscence [23], and this has since then seen intense research, criteria elaborated, and several inner ear disorders defined [11]. However, Snik et al. in 1995 [21], while investigating an air–bone gap in X-linked gusher disorder believed that a dilated internal auditory meatus, the incomplete partition type III and an incomplete bony fundus of the IAM in X-linked gusher led to an abnormal communication between the subarachnoid space (cranial cavity) and the perilymphatic space (inner ear) that defines a third window.

The third-window effect was later detailed by Merchant et al. in 2008 [24]. They attributed the third window-effect due to the shunting of acoustic energy from the middle ear by the third window and propagation of the cochlear travelling wave from the oval to the third window, leading to a rise in air conduction thresholds and a lowering of bone conduction thresholds, resulting in the appearance of an air–bone gap. Anatomical third windows spare the cochlea; for example, the bony canal dehiscence group does not affect cochlear function. However, third windows with cochlear dysplasia affect cochlear function [11]. Therefore, in such an instance, there will be an added cochlear element to the third-window conductive element generating the typical mixed pattern, as in DFNX2. As a result of the third window, the cochlear element also shows fluctuation when measured with bone conduction, as observed in our siblings. In these subjects, there may be absent otoacoustic emissions reflecting the sensory hearing loss, but usually, at least one sided stapedial reflex is preserved unless profound sensory hearing loss with intact tympanometry [25]. Both the siblings in this series demonstrated mixed hearing loss, absent otoacoustic emissions and stapedial reflexes were bilaterally preserved except in one sibling where stapedial reflex was present only in one ear.

Other typical third-window symptoms are conductive dysacusis, autophony, Hennebert’s and Tullio’s phenomenon, disequilibrium, dizziness/vertigo, tinnitus/pulsatile tinnitus, misophonia and gaze-induced tinnitus [11,14]. It is important to note that these reported symptoms may be difficult to glean from children and may be different in children. This is likely because of the third window on a developing vestibular system and a developing skull that contains the bony otic capsule where the dysplasia is located [26]. The children in this series did not complain of any sound-induced audiovestibular symptoms even when older except autophony in SB. They both had delayed speech and motor development and struggled in their playground activities as toddlers.

For a child with a third-window disorder, their cognitive reaction, emotional reaction, effect on schoolwork and social life and on overall development should be factored in. Once our siblings were made aware of the pathology, they reconciled to their conditions very well to do well in life and develop a positive insight for the future. The observation that characteristic third-window symptoms may be absent in children suggests that the possibility of a third window in a child is not just based on anamnesis but rather on a more holistic and an emerging clinical scenario. Indeed, this holistic approach is quite essential to investigate and then confirm a third window for effective management, avoiding missing the diagnosis.

The mixed hearing loss encountered in X-linked gusher disorder is progressive [13,22]. This is likely due to the continuous third-window effect on the growing cochlear deformity that may lead to a heightened cochlear deficit. The siblings in this series both presented progressive hearing loss. They also presented abnormal audiological behaviour from a very early age, i.e., deficiencies in hearing as observed by their caregivers and a subjective hearing loss when they were old enough to report this. The hearing loss does affect speech perception and acquisition unless treated very early to maximize speech development, which we observed in our series. Both the siblings acquired excellent speech because of this early intervention.

The entire publication spectrum regarding X-linked gusher disorder has investigated the hearing loss and the radiological phenotype in some detail but all but one made no mention of a vestibular phenotype. The first author reported vestibular findings in their study on rare third-window disorders in children with the two cases reported in this study but did not elaborate on the evolution of vestibular function or a SHIMP test [11]. The siblings reported in the present study were followed up for over ten years and vestibular function was objectively quantified along with detailed anamnesis to assess compensated vestibular function. This makes this study quite unique and suggests that the vestibular phenotype should not be overlooked. The authors believe that a vestibular deficit will be due to the chronic third-window effect that is transmitted across the membranous labyrinth due to the abnormal inner ear–intracranial cavity connection in the absence of a structural anatomical defect in vestibular anatomy that has also been proposed elsewhere [27]. VNG examination that entailed eye movements that were spontaneous or provoked with the removal of optic fixation were normal in both children; however, the VST battery eliminating visual fixation and proprioception were abnormal in both children.

The hallmark of diagnosing a third window is a characteristic vestibular-evoked myogenic test (VEMP) that assesses saccular (cervical cVEMP) and utricular (ocular oVEMP) function. Both these tests show elevated amplitudes and lowered thresholds in mobile third windows due to hyperstimulation of the otolith sensors because of the third-window effect on the acoustic energy transmission [26]. Studies are limited to investigate VEMPs in third-window disorders in children [28,29,30,31]. In children, VEMPs can be quite heterogenous and depend on the level of involvement of the utricle and the saccule. A damaged or weak otolith sensor will still be stimulated at the VEMP stimulus but might not return the same amplitude as a normal sensor will. As suggested earlier, due to the chronic third-window effect in X-linked gusher disorder on the vestibular system because of the dysplastic cochlea, the vestibular organs might be inherently weak. Consequently, it is probable that VEMPs in children might not show the typical hyperstimulation parameters that are well-established in the diagnosis of adult third windows and may be absent altogether, especially in this condition [11]. In the current series, cVEMP findings were varied with one child showing normal amplitude on one side and absent amplitude on the other while the other showed significantly reduced amplitudes. The thresholds when measured in the ears that did return an amplitude were lower than normal as per our laboratory norms. This reduction of amplitudes can be considered as indicative of reduced saccular function [32]. It is suggested that thresholds are probably better indicators of diagnosing third windows across all age groups [26] as observed in our siblings establishing the third-window effect. Since studies regarding VEMP norms in children are rather limited, we recommend that individual laboratory-based norms are established to make informed inferences that were considered in this present study, noting that they might be quite different from published adult norms [11].

The video head impulse test (vHIT) has revolutionized vestibular diagnostics in recent times [33]. Normative data may be different from those obtained in adults, and like VEMPs, individual laboratory norms must be established [34]. Low vestibulo–ocular reflex (VOR) gains along with overt and covert saccades indicate vestibular semicircular canal high frequency weakness in all six semicircular canals [33]; however, saccades alone are also important to consider [35], and saccades with normal VOR gain have also been reported in vestibular weakness [36,37]. vHIT studies are limited in third windows, and it has been shown that they can be deranged [27,38]. One sibling in this study had saccades on all canals with low VOR gain in the vertical canals to start with, and the other sibling showed normal gains but with saccades on the lateral canals only in the initial testing. Recovery of VOR gain and reduction and clustering/dispersion of saccades are features of vestibular compensation, indicating that vestibular function may recover over a given period of time [39]. Both showed this recovery pattern when tested three years later after active intervention. This recovery of function was also indicated by a subjective corroboration of adequate balance by the child.

The suppression head impulse test (SHIMP) is a new test in the vestibular test battery that is useful for assessing vestibular compensation and is used as an adjunct to the vHIT [40]. This has been hardly studied in children [41]. This paper reports SHIMP results in X-linked disorder for the first time. Our children in the series demonstrated normal SHIMPs with accepted laboratory-based norms in the second assessment and with normal peak saccadic velocities and asymmetry, suggesting good compensation.

We followed-up with these children for a period of seventeen years, and the older child has transitioned to the adult services formally, while the younger one is still under follow-up. They were fitted early with hearing aids. They underwent regular monitoring of hearing loss and digital amplification adjusted accordingly given the progressive nature of their hearing losses. The older sibling was implanted later on one ear and did not want a second implant while the younger one is doing very well with bilateral hearing aids. The outcome was development of normal speech and communication skills that are reported after early intervention for hearing loss in a child [42]. However, it is equally important to engage parents and significant others in the management algorithm that is not standardized as yet [43]. This family-centred engagement is crucial to maximize the outcome of intervention. The families of the siblings in the current series were engaged in every step of their entire journey, as were their school and facilities for their extracurricular activities. In addition, sensory services were involved for real world and classroom support. As they grew older, they were counselled cognitively by the medical team to reconcile, accept and anticipate their futures with their condition. Both children achieved their creative capabilities and are doing well in their academia. The authors, therefore, stress and emphasize that diagnosing and managing a complex disorder like X-linked gusher disorder is way beyond fitting an implant or a hearing aid and is significantly multidisciplinary and holistic for maximizing a positive outcome.

Similarly, a vestibular weakness is required to be addressed early. Both the siblings underwent a course of vestibular rehabilitation and recovered well with their balance. They received situational counselling as to how to avoid provocation that could lead to vestibular decompensation. They could indulge in playground activities and one child resumed his hobby of dancing.

The limitation of this study is its small number, which of course leaves questions for generalisation. However, we believe that these two cases provide a rather important insight into this very complex disorder. It might be difficult to launch dedicated controlled and blind studies given the rarity of the condition, and thus, snapshots like this yield valuable information. We recommend scrupulous follow-ups and a holistic and cognitive approach (a part of the management algorithm that looks beyond only the illness and assesses the child’s thoughts and feelings that can affect behaviour, how the afflicted child can better cope and reconcile to their condition by dissemination of imparted knowledge by the medical team about the condition, including to the parents/caregivers and significant others also in the process) that is crucial to manage a child.

## 5. Conclusions

X-linked gusher disorder is a rare genetic disorder in children that manifests with an audiovestibular phenotype. Hearing loss is usually a mixed one due to a third-window effect as a result of characteristic bony deformities. Vestibular function hitherto unreported may be weak and can be quantified by objective vestibular tests. The condition is progressive and evolves over a period of time where hearing loss progresses, and vestibular weakness may undergo partial compensation, rendering children symptomatic. Early audiovestibular intervention is pivotal with multidisciplinary engagement in a holistic way involving significant others in the care. This achieves a maximal favourable outcome underpinning the importance of regular follow-ups for these children.

## Figures and Tables

**Table 1:** SA—Hearing symptoms and audiological test results (air and bone conduction thresholds masked whenever necessary and averaged between 500 Hz and 4 kHz in dBHL).

Symptoms	Tymp/AR/OAE/ST	Age (Years)	AC R/L	BC	AB
Hearing difficulties; speech delay	Normal tymp ART absent L OAE absent bil; ST with Manchester Junior Word—in quiet86% at 60 dBA; 76% at 50 dBA; in noise (class babble) 83% at V60/N55.	18	75/NT	52.5	27.5
		14	76.25/NT	51.25	25
		10	75/NT	50	25
		6	81.25/100	48.75	35

Year—Year of test; AC R/L—air conduction thresholds right and left; BC—bone conduction thresholds; AB—air–bone gap; Tymp—tympanometry; AR—acoustic reflex test; bil—bilateral; NT—no threshold detected; note the fluctuation in BC and AB gap that is typical of a third-window disorder; ST—speech test; V60—voice at 60 dBA; N50/55—noise at 50/55 dBA.

**Table 2:** SA—vestibular/balance symptoms and vestibular function tests (VNG and VEMP).

Symptoms	VNG/VST/SVV, Age 12 and 15 Years	cVEMP AA/RA R μV	cVEMP AA/RA L μV	Asymmetry	cVEMP Threshold dBnHL Age 15 Years
Delayed motor development; unsteadiness; postural instability; difficult ambulation in darkness; difficulty in reading; difficulty in playground activities; absent Tullio’s or Hennebert’s phenomenon	Normal; abnormal VST	92.5/2.9	Nil	100%	75 (R)

VNG—Videonystagmography without optic fixation assessing head shake, head heave, ocular counter rolling and presence or absence of any spontaneous or provocation nystagmus and smooth pursuits/saccades; VST—static posturography in different conditions; SVV—subjective visual vertical; AA—absolute amplitude; RA—rectified amplitude; R—right; L—left.

**Table 3:** SA—video head impulse test and suppression head impulse test.

Year	VOR L/R ASCC	VOR L/R LSCC	VOR L/R PSCC	Saccades	SHIMP
Age 12 years	0.41/0.3	0.72/0.88	0.54/0.64	All canals dispersed	
Age 15 years	0.5/0.69	0.7/0.84	0.87/0.59	Only in left lateral and right anterior, some clustering	241–266°/s PSV L and 243–306°/s PSV R; 5% asymmetry

VOR L/R—VOR gain left and right; ASCC—anterior or superior semicircular canal; LSCC—lateral semicircular canal; PSCC—posterior semicircular canal; PSV—peak saccadic velocity of SHIMP saccades; note the improvement of VOR gain with time; R—right; L—left.

**Table 4:** SB—hearing symptoms and audiological test results (air and bone conduction thresholds masked whenever necessary and averaged between 500 Hz and 1- kHz in dBHL).

Symptoms	Tymp/AR/OAE/ST	Age (Years)	AC R/L	BC	AB
Hearing difficulties; speech delay	Normal tymp ART present bil OAE absent bil ST with Manchester Junior Word—in quiet 86% at 50 dBA; 40% at 40 dBA; 60 dBA speech with 50 dBA; in noise (class babble) 83% at V60/N55	16	81.25/83.3	51.25	27.5
		12	78.75/78.75	53.75	22.5
		8	76.25/75	48.75	27.5
		4	87.5/85	27.5	56.25

Year—year of test; AC R/L—air conduction thresholds right and left; BC—bone conduction thresholds; AB—air–bone gap; Tymp—tympanometry; AR—acoustic reflex test; bil—bilateral; note the fluctuation in BC and AB gap that is typical of a third-window disorder; ST—speech test; V60/N55—voice at 60 dBA; N55—noise at 55 dBA.

**Table 5:** SB—vestibular/balance symptoms and vestibular function tests (VNG and VEMP).

Symptoms	VNG/VST/SVV Age 10 and 13 Years	cVEMP AA/RA R μV	cVEMP AA/RA L μV	Asymmetry	cVEMP Threshold dBnHL Age 13 Years
Delayed motor development; unsteadiness; postural instability; autophony but absent Tullio’s or Hennebert’s phenomenon	Normal; abnormal VST	57.3/1.1	58.8/0.8	15%	75 (R + L)

VNG—videonystagmography without optic fixation assessing headshake, head heave, ocular counter rolling, mastoid vibration and presence or absence of any spontaneous or provocation nystagmus and smooth pursuits/saccades; VST—static posturography in different conditions; SVV—subjective visual vertical; AA—absolute amplitude; RA—rectified amplitude; R—right; L—left.

**Table 6:** SB—video head impulse test and suppression head impulse test.

Age in Years	VOR L/R ASCC	VOR L/R LSCC	VOR L/R PSCC	Saccades	SHIMP
10	0.66/0.59	0.88/0.92	0.65/0.72	Only laterals dispersed	
13	0.68/0.8	1.07/0.95	0.95/0.69	Only lateral R clustered	287–358°/s PSV L and 342–414°/s PSV R; 4% asymmetry

VOR L/R—VOR gain left and right; ASCC—anterior or superior semicircular canal; LSCC—lateral semicircular canal; PSCC—posterior semicircular canal; PSV—peak saccadic velocity of SHIMP saccades; note the improvement of VOR gain with time; R—right; L—left.

## Data Availability

All data are presented in article.
